# Editorial

**Published:** 2014-07-28

**Authors:** Shahina Tabassum

**Affiliations:** Professor and Chairman Department of Virology Bangabandhu Sheikh Mujib Medical University (BSMMU) Shahbag, Dhaka-1000, Bangladesh Phone: +8802 8617099 e-mail: shahina.tabassum@yahoo.com

**Genetic Diversity of Hepatitis B Virus: Will It influence HBV Vaccination?**

Albeit the availability of a safe and effective vaccine for hepatitis B virus (HBV) since 1982 and worldwide vaccination policies, infection with HBV remains a global public health issue causing more than 240 million chronic infections and more than 780,000 deaths per year.^1^ HBV infection can manifest as acute/fulminant hepatitis or various chronic conditions, including inactive carrier state, chronic hepatitis, cirrhosis and hepatocellular carcinoma (HCC).^2^ It has been encouraging to witness the recent discoveries in HBV infection with insights into the existence of genotypes, subtypes, mutant variants, knowledge regarding host, viral and environmental factors on the disease course as well as advances in new generation of vaccines and treatment modalities.

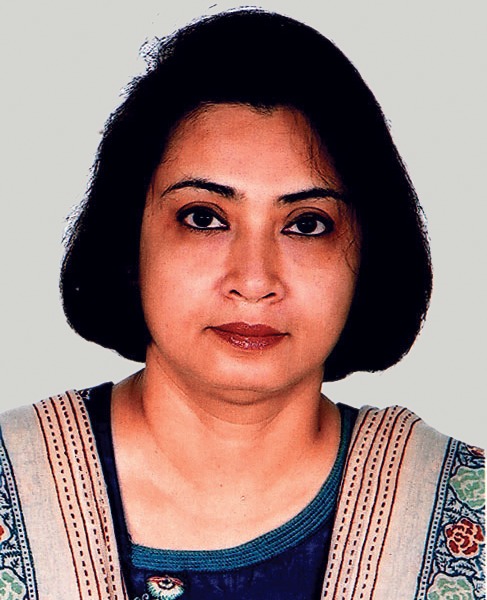



Hepatitis B virus is a small dsDNA virus belonging to the family Hepadnaviridae with a genome size of only 3,200 nucleotides. The partially double-stranded circular DNA contains four overlapping open reading frames encoding the S (surface), C (core), P (polymerase) and × genes. The S protein or hepatitis B surface antigen (HBsAg) is the target of the protective humoral immune response in humans induced by vaccination. The HBV genome evolves quickly over time because the viral reverse transcriptase has a high error rate with an estimated 10^-4^ to 10^-6^ nucleotide substitutions per site per year.^3^ This unique replication strategy is responsible for the majority of the point mutations, deletions and insertions observed in the HBV genome. The long-time evolution of HBV has led to the occurrence of various genotypes, subgenotypes, mutants, recombinants and even quasispecies of HBV.^4^ The genetic variability in association with human migration has caused the divergence of HBV strains into distinct geographic distribution and different biological properties affecting the clinical outcome of HBV disease.

Analyses of the divergence of HBV genomic sequences have identified eight confirmed (A-H) and two provisional (I and J) HBV genotypes and several subgenotypes (subtypes), based on more than 8% divergence over the complete genome for separate genotypes, 4 to 8% for subgenotypes and less than 4% nucleotide diversity for further divergence into ‘clades.’^5^ Genotype A appears to be the predominant genotype in Europe, Africa and North America; at least seven subgenotypes have been defined within HBV genotype A; subgenotype A1 is prevalent in Africa and Asia, A2 in Europe and the USA, and A3-A7 is prevalent in various countries of Africa. Genotypes B and C are more prevalent in the Asia-Pacific region; genotype B has nine subgenotypes: B1 was reported from Japan, while B2-B9 from different areas of Southeast Asia. Similarly, genotype C has been classified into six subgenotypes, with characteristic geographical distributions. Subgroup C1 was isolated from Taiwan, while C2-C16 were commonly found in East Asian countries, like China, Oceania, Australian Aborigines, Philippines and Indonesia. Genotype D is ubiquitous and distributed globally, especially among intravenous drug users; subgenotype D1 is prevalent in Pakistan, Iran, and its neighboring countries, D2 in Eastern Europe and Russia, D3 in Siberia, South Africa and Alaska, D4 in Oceania and Somalia, while D5-D9 were reported from India, Indonesia, Tunisia and Nigeria. Genotype E is found in West Africa and in the Afro-Colombian population. Genotype F, which is classified into subgenotypes F1-F4, is predominant in several South American countries. Genotype G is prevalent in France, Germany, Mexico and the United States of America, while genotype H is found in Mexico and Central America. Genotype I was reported from Vietnam and Laos, and genotype J was detected from Japan.^6^

Recombination in HBV is principally the result of the coinfection of a host with more than one strain from different (sub) genotypes. Recombinations may occur between strains of different subgenotypes of the same genotype or between strains of different genotypes. Currently, more than 30 recombinants have been described. Most recombinant strains are reported from regions where the prevalence of HBV infection is high and prophylaxis and control of infection is low. HBV variants originating from mutations and mutant selections have medical and public health relevance. Since HBV replicate via an error-prone viral reverse transcriptase, it results in a large pool of quasispecies with mutations spread throughout the genome. The precore/core promoter mutants and pre S deletion mutants are selected during the natural course of chronic HBV infection. With efforts to control HBV infection through vaccination and antiviral therapy, novel-resistant mutants have emerged under the pressure of neutralizing antibody responses, leading to vaccine resistance and resistance to immunotherapy.^7^ Previously, that is, before molecular analyses into genotype, subgenotype and clades, HBV strains were classified into antigenic subtypes based on the antigenic properties of its major surface glycoprotein the HBsAg. The ‘a’ determinant region is common to all serotypes of HBV, with at least four subdeterminants, d or y and r or w. On the basis of different potential combinations of the above allelic variations, HBV serotypes are divided into adr, adw, ayr or ayw, with further division into a total of 10 serological subsubtypes. This serotype-based classification is still used, and epidemiological studies indicate relationships between serologic subtypes and genotypes.^5^

The natural history of chronic hepatitis B differs between HBV genotypes with regard to progression to liver fibrosis and development of HCC.^8^ Some studies suggest that genotypes B-D are related to poor clinical outcomes. Furthermore, HBV genotypes differ in their response to antiviral treatment, for example susceptibility to interferon-a is greater in HBV genotype A-infected patients than in those infected with genotypes D, B or C, but the response to treatment with nucleoside/ nucleotide analogs is somewhat independent of HBV genotypes and may probably influence vaccination efficacy.^9^ Globally, considerable progress has been made in HBV prevention. Various studies have demonstrated significant decrease of HBV infection, chronic carriage and HBV-related complications, including HCC, after the introduction of universal infant HBV vaccination. It is anticipated that increasing HBV vaccination coverage worldwide will have positive impact on HBV eradication in the near future. The currently available genetically engineered HBV vaccines are derived from genotypes A and D of HBV. In regions where other genotypes are prevalent, clinical vaccine effectiveness findings demonstrate that there is significant cross-protection against other genotypes provided by the currently used HBV vaccines. Notwithstanding its success in most cases, there are many reports of vaccination failures because of genotype complexities.^9^ Development of infections by other genotypes among vaccinated individuals and S gene variations isolated from vaccine failure cases have also been reported in various studies.^10,11^ These examples of vaccine failures suggest that, in future, it is important to assess the protection against chronic infection and disease by different genotypes and HBV variants.

Regardless of the worldwide diversity of HBV genotypes, significant reductions in the disease burden have resulted in global prevention of HBV. Prevention of infection with the effective HBV vaccines, new treatment options and lifestyle practices has reached remarkable levels in developed countries, while continuing efforts to improve and implement mass vaccination programs are expected to produce comparable outcome in resource-limited countries. Still, much remains to be understood about the genetic diversity of HBV and their impacts on the current vaccination coverage, clinical course and long-term outcome of hepatitis B infection. It is important to determine whether vaccine strategies should be adapted in relation with the genotypes that are more prevalent in a given population and if efforts should be directed toward developing vaccines that protect against all the genotypes of HBV. Hence, further research and understanding of the effects of vaccination on genotype distribution is necessary to expand our knowledge regarding the effectiveness of currently used HBV vaccines. Nonetheless, focus of such studies should concentrate on viral, host and other environmental factors that may play vital role in achieving effective vaccination coverage.

**Shahina Tabassum**

Professor and Chairman

Department of Virology

Bangabandhu Sheikh Mujib Medical University (BSMMU)

Shahbag, Dhaka-1000, Bangladesh

Phone: +8802 8617099

e-mail: shahina.tabassum@yahoo.com
